# Need for Improvement in Death Pronouncements in Palliative Care Units

**DOI:** 10.1089/pmr.2023.0053

**Published:** 2023-12-22

**Authors:** Jun Hamano, Kento Masukawa, Satoru Tsuneto, Yasuo Shima, Tatsuya Morita, Yoshiyuki Kizawa, Mitsunori Miyashita

**Affiliations:** ^1^ Department of Palliative and Supportive Care, Institute of Medicine, University of Tsukuba, Tsukuba, Ibaraki, Japan.; ^2^ Department of Palliative Nursing, Health Sciences, Graduate School of Medicine, Tohoku University, Sendai, Miyagi, Japan.; ^3^ Department of Human Health Sciences, Graduate School of Medicine, Kyoto University, Sakyo-ku, Kyoto, Japan.; ^4^ Hospice Palliative Care Japan, Ashigarakami, Kanagawa, Japan.; ^5^ Department of Palliative and Supportive Care, Seirei Mikatahara General Hospital, Hamamatsu, Japan.; ^6^ Research Association for Community Health, Hamamatsu, Japan.

**Keywords:** bereaved family, death pronouncement, palliative care units

## Abstract

**Background:**

Death pronouncement is an important moment that can impact a family's bereavement process; however, necessary improvements in physicians' behavior during death pronouncement remain unclear.

**Objectives:**

To explore whether the lack of certain behaviors by the physician was associated with a perceived need for improving death pronouncement for advanced cancer patients in palliative care units (PCUs).

**Methods:**

This study was a secondary analysis of a nationwide multicenter questionnaire survey conducted in 2018 that targeted bereaved family members of cancer patients who died in PCUs. We performed univariate analysis to investigate the need to improve behavior toward death pronouncement. We performed bivariate analysis to investigate the relationship among the need for improvement in behavior toward death pronouncement, physician attribution (primary responsible physician, a member of the same team, and another physician), and nine specific behaviors.

**Results:**

Four hundred twenty-two questionnaires (64.2%) were returned. We analyzed 356 responses and found that 32.5% perceived the need to improve death pronouncement. Lack of certain behaviors at death pronouncement, especially not explicitly explaining the cause of death to family members (odds ratio: 11.89, *p* < 0.001), were positively associated with the need for improvement. There were significant differences among the types of physician attribution regarding the need for improvement (primary responsible physician vs. a member of the same team vs. another physician [15.1% vs. 42.6% vs. 45.7%, *p* < 0.001]).

**Conclusion:**

There was a significant positive association between the lack of certain behaviors toward death pronouncement and the need for improvement. The major lack of behavior toward death pronouncement was not explicitly explaining the cause of death to family members and not calling out to the patient before beginning the patient's examination.

## Key Message

We performed a secondary analysis of a nationwide survey. In this study, we aimed to explore the association between physician's behavior and improvement of death pronouncement in palliative care units. Our findings suggest that the need for improvement in death pronouncement was associated with specific behaviors during the pronouncement.

## Introduction

The essential role of palliative care is to alleviate the distress of the patient and their family.^1^ The bereavement of a loved one is a traumatic event, and it may affect the mental health of the bereaved family.^2–4^ The processes and the circumstances around bereavement influence its impact on families and death pronouncement is one of the most influential moments in the bereavement process. Shinjo et al. reported that a bereaved family’ experience around the death pronouncement had an effect on their emotional distress.^5^

Several statements and recommendations include appropriate behavior toward death pronouncement and describe it as an essential skill that physicians should acquire.^6–10^ Kusakabe et al. reported several behaviors of physicians, such as physicians not clearly verifying the time of death, that were significantly positively correlated with family perceived need for improvement in the home care setting.^11^ However, one of the limitations of this study was that it was conducted in the home care setting in which the primary responsible physician usually performs death pronouncement.

Hatano et al. conducted a cross-sectional questionnaire survey of bereaved caregivers who had lost a family member in a palliative care unit (PCU) to examine their perception of physician's behavior toward death pronouncement.^12^ They reported that caregivers whose family member's death was confirmed by the primarily responsible physician were significantly more satisfactory than those whose family member's death was confirmed by an unfamiliar physician. However, they also reported that 82% of caregivers did not think it mattered whether it was the primary responsible physician who confirmed the death. Thus, further investigation of the potential mechanisms behind desirable physician behaviors during death pronouncements are needed that consider the type of physician involved.

We previously conducted a video-vignette study to explore the components of a physician's behavior that affect participant-perceived physician compassion.^13^ We found that four components (explaining that the physician on-call had been called out; performing a respectful examination; ascertaining the time of death with a wristwatch; and reassuring the family that the patient did not experience pain) resulted in higher levels of participant-perceived physician compassion. On the other hand, the component “waiting until the family members calm themselves down” was not perceived as a compassionate behavior by participants.^13^ However, as our previous study was based on hypothetical scenarios, it is necessary to use real-world data.

We aimed to explore whether a lack of certain behaviors by the physician and types of physician attribution were associated with the need for improving death pronouncement for advanced cancer patients in PCUs. In addition, we aimed to explore the association between the type of physician attribution and major depressive disorder/complicated grief of the bereaved families.

## Methods

This study was a secondary analysis of a nationwide multicenter questionnaire survey targeting the bereaved family members of cancer patients who died in PCUs, general wards, or at home to evaluate the quality of end-of-life care in Japan (Japan Hospice and Palliative Care Evaluation Study 4: J-HOPE 4 study). We recruited potential participants from 187 PCUs, 14 general hospitals, and 14 home care facilities that were members of Hospice Palliative Care Japan before January 31, 2018. Bereaved family members who had been bereaved for at least three months were included in the study.

### Participants and procedures

A cross-sectional, anonymous, self-reported questionnaire survey was conducted in 2018. We asked each PCU to identify and list up to 80 bereaved family members of patients who died before January 31, 2018. The major inclusion criteria were that the patient was 20 years or older and died of cancer. The major exclusion criterion was that the candidate participant had severe psychological distress determined by the primary care physician and nurses.^14^ Questionnaires were sent to the bereaved family members identified by each participating PCU, along with an explanation of the survey. The return of the completed questionnaire to the study secretariat office (Tohoku University) within one month was regarded as consent to participate in the study. In addition, we sent a reminder to nonresponders one month after sending the questionnaire. If they did not wish to participate in the study, they were asked to check a “no participation” box and return the incomplete questionnaire. The Institutional Review Board of Tohoku University (2017-2-236-1) and those of all participating PCUs approved the protocol of this study.

### Participant characteristics

We asked the primary responsible physicians in charge of treatment for the patient to collect background characteristics (age, sex, primary tumor site, and duration of admission) of each patient via medical records. In addition, the bereaved family members were asked for details concerning their age, sex, relationship with the patient, educational background, and whether or not the bereaved family members who responded to the questionnaires were present during death pronouncement.

### Measurements

We asked, “How much improvement do you think is necessary for death pronouncement by the physician?” according to the methodology of previous studies.^5,11,15,16^ The response was rated on a 6-point scale ranging from 1 (much improvement needed) to 6 (no improvement needed at all). Then, to explore the factors that potentially contributed to a family perceived need for improvement, we asked about nine specific behaviors toward death pronouncement performed by physicians based on our previous randomized, scripted video-vignette study and other previous studies. We used binary categorical variables (I think so/I do not think so).^11–13^

In addition, we asked bereaved families the attributions of the physician who pronounced the death using three categorical variables (primary responsible physician, a member of the same team, another physician).

### Statistical analysis

Sociodemographic data from deceased patients and bereaved families were summarized using descriptive statistics. We divided the 6-point scales of the need for improvement in death pronouncement into binary categorical variables and divided patients into two groups: a score of 1 (much improvement needed) to 4 (slight improvement needed) into an improvement needed group; and a score of 5 (little improvement needed) and 6 (no improvement needed at all) into a no improvement needed group).

The validity of the Japanese version of the Patient Health Questionnaire-9 has been confirmed.^17^ We used it to measure depression among the participants. Responses were rated on a scale from 0 to 3, with total scores ranging from 0 to 27. A total score of ≥10 indicates that the respondent is likely to develop major depressive disorder.^18^

We also used the Brief Grief Questionnaire (BGQ) to measure the grief of the participants, which is composed of five items that are rated using a 3-point Likert scale, with a higher score representing a more severe grief reaction.^19^ A total score of ≥8 indicates that the respondent is likely to develop complicated grief. A previous study confirmed the validity of the Japanese version of the BGQ in the general Japanese population.^20^

We performed a univariate analysis to assess the association between physician attributes and their behavior during death pronouncement using the Kruskal–Wallis test. We also conducted bivariate analysis to examine the need for improvement in death pronouncement in relation to the type of physician attribution (primary responsible physician, a member of the same team, another physician) and each of the nine behaviors. In addition, we conducted multivariate logistic analysis for major depressive disorder and complicated grief of the bereaved family. Based on previous research and discussion among authors,^11,12^ we selected the variables for bivariate logistic analysis, which included the type of physician attribution and each of the nine behaviors. Significance was *p* < 0.05, and all analyses were carried out using SPSS-J software (version 28.0; IBM, Tokyo, Japan).

## Results

Six hundred fifty-seven family members met the inclusion criteria, and 422 (64.2%) responded. Among the responses, 66 family members refused to participate; therefore, we analyzed 356 responses (84.4%). Characteristics of the participants are summarized in Table 1. The mean age of the patients who died of cancer was 72.2 ± 12.2 years, and 53.7% were men. The most frequent primary tumor was lung cancer, followed by hepatobiliary/pancreatic cancer and stomach/esophagus. The duration of admission was 27.1 ± 28.6 days. Bereaved family members had a mean age of 61.8 ± 12.5 years, and 34.6% were men. The bereaved person was most frequently the husband/wife of the patient, followed by a child of the patient. The time between the patient's death and caregiver survey response was 407.5 ± 101.9 days. Three hundred twenty-four family members (91.0%) said they were present during the death pronouncement (Table 1).

**Table 1.** Backgrounds

	All (***n*** = 356)	%
Patients		
Age (mean ± standard deviation)	72.2 ± 12.2	
Sex		
Male	191	53.7
Female	165	46.3
Primary tumor sites		
Lung	65	18.3
Liver, bile duct, pancreas	55	15.4
Stomach, esophagus	55	15.4
Colon, rectum	38	10.7
Head and neck, brain	29	8.1
Prostate, kidney, bladder	24	6.7
Breast	24	6.7
Uterus, ovary	24	6.7
Blood	11	3.1
Others	31	8.7
Length of admission or care at home (days)	27.1 ± 28.6	
Bereaved family members		
Age (mean ± standard deviation)	61.8 ± 12.5	
Sex		
Male	123	34.6
Female	228	64.0
Relationship with patient		
Husband/wife	176	49.4
Child	131	36.8
Daughter-in-law or son-in-law	10	2.8
Parents	11	3.1
Siblings	18	5.1
Others	6	1.7
Education		
Less than high school	32	9.0
High school graduate	147	41.3
Posthigh school education	168	47.2
Caregiver's physical health status at the last admission		
Good	95	26.7
Moderate	195	54.8
Not good	51	14.3
Bad	13	3.7
Caregiver's mental health status during the last admission		
Good	48	13.5
Moderate	158	44.4
Not good	107	30.1
Bad	36	10.1
Duration of bereavement (mean ± standard deviation, days)	407.5 ± 101.9	
Present during death pronouncement	324	91.0
Attribution of physician who performed the death pronouncement		
Primary responsible physician	152	42.7
A member of the same team	47	13.2
Another physician	116	32.6

Among the bereaved families, 32.5% perceived the need for improvement in death pronouncement (1: much improvement needed to 4: slight improvement needed). Comparing the prevalence of the need for improvement in death pronouncement by physician attribution revealed significant differences among the three physician groups (primary responsible physician, a member of the same team, another physician [15.1% vs. 42.6% vs. 45.7%, *p* < 0.001]).

Table 2 shows the association among the physician's attribution and behaviors around death pronouncement. About 80% of physicians introduced themselves before death pronouncement and checked pupils for response to light. Comparing the frequency of physician behavior toward death pronouncement by physician attribution revealed significant differences among the three physician attributions; however, this was not found for checked pupils for response to light.

**Table 2.** Association Between Attribute of Physician and Physician Behavior Toward the Death Pronouncement

Item	All (***n*** = 324)	%	Primary responsible physician (***n*** = 152)	%	A member of the same team (***n*** = 47)	%	Another physician (***n*** = 116)	%	***p*** ^*^
Checked pupils for response to light	267	82.4	130	85.5	37	78.7	94	81.0	0.078
Introduced him/herself before death pronouncement	260	80.2	134	88.2	40	85.1	80	69.0	<0.001
Confirmed the relationships of persons present before death pronouncement	212	65.4	128	84.2	32	68.1	47	40.5	<0.001
Confirmed that all important family members were present before death pronouncement	186	57.4	110	72.4	28	59.6	43	37.1	<0.001
Checked for lung and heart sounds by stethoscope	183	56.5	98	64.5	26	55.3	56	48.3	0.031
Called out to the patient before beginning his/her examination	176	54.3	102	67.1	27	57.4	45	38.8	<0.001
Expressed empathy to family members	140	43.2	93	61.2	25	53.2	19	16.4	<0.001
Reviewed the death certificate with the family	129	39.8	73	48.0	22	46.8	30	25.9	<0.001
Explained the cause of death to the family members explicitly	126	38.9	87	57.2	17	36.2	17	14.7	<0.001

^*^ Kruskal–Wallis test.

Table 3 shows the bivariate logistic analysis with types of physician's attribution for the need for improvement in death pronouncement. There was a significant positive association between the lack of certain behaviors toward death pronouncement and the need for improvement. The major lack of behavior toward death pronouncement was not explicitly explaining the cause of death to family members and not calling out to the patient before beginning the patient's examination. Multivariate logistic analysis for major depressive disorder and complicated grief of the bereaved family revealed that there was no significant association with type of physician attribution (Appendix Tables A1 and A2).

**Table 3.** Bivariate Logistic Analysis for the Need for Improvement in Death Pronouncement

	Odds ratio	95% CI	***p***	Nagelkerke ***R***^2^
Not introduced him/herself before death pronouncement	4.61	1.98–10.72	<0.001	0.208
Not identifying the relationships of persons present before death pronouncement	4.44	2.20–8.96	<0.001	0.233
Not confirming that all important family members were present before death pronouncement	4.35	2.32–8.15	<0.001	0.255
Not calling out to the patient before beginning his/her examination	9.34	4.67–18.68	<0.001	0.387
Not checking pupils for response to light	3.03	0.92–9.97	0.069	0.188
Not checking for lung and heart sounds by stethoscope	2.76	1.37–5.55	0.004	0.277
Not explaining the cause of death to the family members explicitly	11.89	5.25–26.92	<0.001	0.397
Not expressing empathy to family members	7.87	3.84–16.15	<0.001	0.370
Not reviewing the death certificate with the family	4.27	2.26–8.05	<0.001	0.294

CI, confidence interval.

## Discussion

This study provides insights into factors that are associated with family members' perceptions of needed improvements toward death pronouncement, including the type of physician conducting the pronouncement and specific behaviors the physician performs. A strength of this study is that it includes more patients than previous similar studies.^11,12^

The most important finding was that lack of certain behaviors at death pronouncement, such as not explicitly explaining the cause of death to family members, were positively associated with the need for improvement in death pronouncement in all three types of physician attribution. This result is partially consistent with our previous video-vignette study, which found that “waiting until the families calm themselves down” and “performing examination respectfully” were associated with significantly higher perceived physician compassion.^13^ On the other hand, our result is inconsistent with a previous study conducted in the home care setting by Kusakabe et al.^11^ that reported that identifying the relationships of persons present, confirming that all important family members were present, explaining the cause of death to the family members explicitly, and expressing empathy to family members were not significantly associated with the family perceived need for improvement in behaviors toward death pronouncement by univariate analysis.^6^ A possible reason for this difference may be that Kusakabe et al.^11^ did not include physician's attributes. In the home care setting, the primary responsible physician or an acquainted physician gives the death pronouncement; therefore, it would have been challenging to analyze physician's attributes. Hence, our results are meaningful in the hospital setting, where another physician often confirms death pronouncements; however, they may be challenging to apply to the home setting. Although our result indicated certain physician behaviors are important toward death pronouncement, some behaviors may be difficult to perform by another physician such as explaining the cause of death to the family members explicitly and expressing empathy to family members. Further studies are needed to identify the essential behaviors in terms of types of physician attribution.

A possible contributing factor to the need for improvement in death pronouncement is the lack of education for physicians and medical students regarding death pronouncement. The nine specific behaviors in death pronouncement by physicians we investigated in this study may be encompassed by the recently proposed list of competencies and entrustable professional activities for resident physicians during death pronouncement.^21^ Thus, our findings could be used in educational programs for medical students and physicians toward death pronouncement.

The second important finding was that there were significant differences regarding the frequency of physician behavior toward death pronouncement among the physician attributions. Our findings support a previous study by Hatano et al. that reported that caregivers were less satisfied with an unfamiliar physician performing a death pronouncement than the primary responsible physician.^12^ Our bivariate logistic analysis results suggest that the cause of the reduced satisfaction reported in Hatano et al.^12^ may not only have been that another physician performed the death pronouncement but also that the other physician had a lack of consideration for the family. Both our study and Hatano et al.^12^ were cross-sectional questionnaires of bereaved caregivers who had lost a family member in a PCU, but the purpose of the study was different: caregivers' perception of satisfaction and the need for improvement with death pronouncement. Therefore, the outcome measurement scale and variables were different. In addition, although both studies assessed physician's attribution, our study categorized the types of physician's attribution into three groups, whereas Hatano et al.^12^ categorized into two groups. Thus, our study classifies physician types more in line with the reality of clinical practice and we believe the results are transferrable to clinical practice. Thus, our study result adds a new key message for the behavior of the death pronouncement; when physicians who are not generally involved in patient care perform the death pronouncement, they need to say or do specific things such as explaining the cause of death to the family members explicitly and expressing empathy.

Our study had several limitations. First, we could not exclude recall bias due to the retrospective design. However, several previous studies performed 3 to 12 months after the patient's death suggested that this interval is reasonable considering both recall bias and the grieving process.^22–25^ Second, the instrument was based on a literature review and expert opinions. The validity or reliability of this measure was not statistically investigated; however, several previous studies used a part of this instrument. Other unmeasured factors could be related to the need for improvement in physician behavior toward death pronouncement. Third, participants were restricted to those who had lost a family member with advanced cancer in a PCU; therefore, our findings may not extend to other populations such as noncancer patients. Further research is needed to evaluate death pronouncement in the care setting for noncancer patients. Fourth, bereaved families with psychological distress may not have returned the questionnaire, although the response rate of this study was higher than that of a previous study.^26^ Therefore, the prevalence of depressive mood and complicated grief among bereaved families in a PCU may have been underestimated.

## Conclusion

There was a significant positive association between the lack of certain behaviors toward death pronouncement and the need for improvement. The major lack of behavior toward death pronouncement was not explicitly explaining the cause of death to family members and not calling out to the patient before beginning the patient's examination. Our findings suggest that physicians should pay attention to their behavior toward death pronouncement and develop behaviors that will have a positive impact on the bereaved family.

## Data Statement

The data that support the findings of this study are available on request from the corresponding author. The data are not publicly available due to privacy or ethical restrictions.

## Abbreviations Used

BGQ: Brief Grief Questionnaire
CI: confidence interval
PCUs: palliative care units

## Appendix

**Appendix Table A1.** Multivariate Logistic Analysis for Major Depressive Disorder of Bereaved Family

	Odds ratio	95% CI	***p***
Attribution of physician who performed the death pronouncement: Primary responsible physician			0.454
A member of the same team	1.34	0.49–3.64	0.568
Another physician	1.56	0.77–3.16	0.213
Bereaved family member's age	0.99	0.96–1.03	0.245
Relationship with patient: Others			0.086
Child	4.00	0.86–18.59	0.077
Husband/wife	1.75	0.35–8.69	0.494
Caregiver's physical health status at the last admission	2.23	1.06–4.69	0.035
Caregiver's mental health status at the last admission	2.41	1.18–4.92	0.016

CI, confidence interval.

**Appendix Table A2.** Multivariate Logistic Analysis for Complicated Grief of Bereaved Family

	Odds ratio	95% CI	***p***
Attribution of physician who performed the death pronouncement: Primary responsible physician			0.301
A member of the same team	0.68	0.18–2.58	0.570
Another physician	1.60	0.75–3.42	0.223
Bereaved family member's age	0.98	0.95–1.01	0.245
Relationship with patient: Others			0.086
Child	4.00	0.86–18.59	0.077
Husband/wife	1.75	0.35–8.69	0.494
Caregiver's physical health status at the last admission	2.23	1.06–4.69	0.035
Caregiver's mental health status at the last admission	2.41	1.18–4.92	0.016
